# Synthesis of Nanoparticles and Theoretical Model of Their Retention in Plasma of RF Capacitive Discharge with Vertically Arranged Electrodes in Acetylene

**DOI:** 10.1021/acsomega.2c05846

**Published:** 2022-12-15

**Authors:** Valeriy Lisovskiy, Alexey Minenkov, Stanislav Dudin, Sergiy Bogatyrenko, Pavel Platonov, Vladimir Yegorenkov

**Affiliations:** † School of Physics and Technology, V.N. Karazin Kharkiv National University, Kharkiv61022, Ukraine; ‡ Christian Doppler Laboratory for Nanoscale Phase Transformations, Center for Surface and Nanoanalytics, Johannes Kepler University Linz, Linz4040, Austria

## Abstract

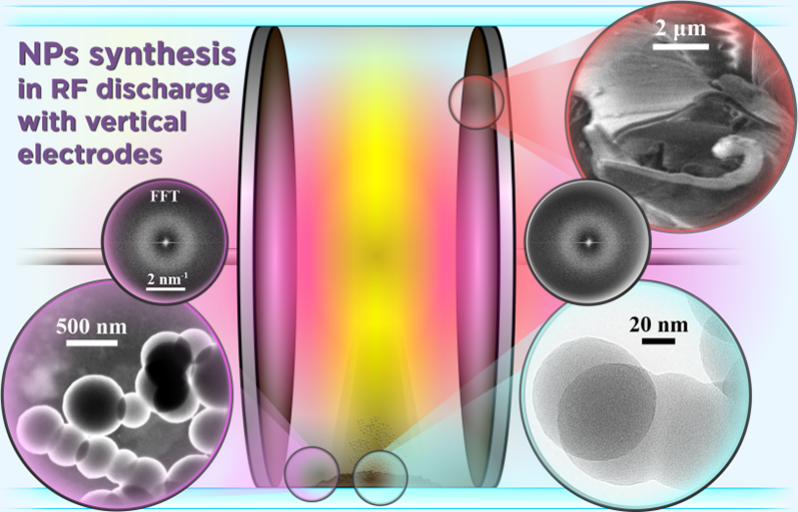

In the present research, experiments on the formation and retention of nanoparticles (NPs) in the plasma of radio frequency (RF) capacitive discharge in acetylene were carried out with vertically positioned internal electrodes. It has been shown via SEM and TEM techniques that NPs found on the horizontal tube wall after the discharge operation have a spherical shape with a predominant diameter of approximately 400–600 nm. HRTEM analysis reveals their amorphous structure. At the same time, such NPs were not found on vertical electrodes, only a polymer film was deposited. To elucidate the possibility of NPs leaving the plasma in the direction of vertical electrodes, a model of NP retention in the near-electrode sheath of an RF capacitive discharge was elaborated. The model has shown that nanometer- and even micrometer-sized particles formed in the plasma cannot cross the near-electrode sheath and reach the electrode surface. For the plasma consisting of three charged components (positive ions, electrons, and NPs), an analytical model of ambipolar diffusion was developed. Applying this model, it has been shown that the ambipolar electric field can keep the micrometer-sized NPs in the plasma if their concentration is low. However, in the case of a high concentration of NPs, they can be retained with a diameter of no more than a few hundred nanometers due to a significant decrease in the ambipolar electric field. The calculation results are in agreement with our experimental data.

## 1 Introduction

Nanoparticles (NPs) are part of cosmic dust clouds and are also often observed in laboratory plasma. Considerable attention is paid to dusty plasma and the processes occurring in it. In particular, the formation and properties of NPs in technological plasma are of principal interest. Such NPs can appear during the deposition of carbon-containing films and be a part of them falling on the surface over the process. Nanoinclusions embedded in a polymer film can change its properties.^1,2^ Sometimes such insertions are useful, for instance, in a number of microdevices, e.g.^3^ However, very often the presence of NPs in the volume of a polymer film is unacceptable. For instance, polyacetylene and diamond-like carbon (DLC) coatings obtained in the plasma of a radio frequency (RF) capacitive discharge in acetylene, can be utilized for biomedical applications or chemical reactors. In this case, such a film plays a protective role, and the presence of nanoinclusions and/or other inhomogeneities can have a detrimental effect.^4^

Polymeric nanomaterials obtained from acetylene (polyacetylene and its derivatives) can be dielectric or conductive^5^ and are widely used for advanced energy storage,^6,7^ flexible electronics^8,9^ and for biomedical applications,^10^ etc. Note that not only nanostructured films have found application, but also NPs of polyacetylene and its derivatives, a review of which is given in^11^ (circuits, sensors, drug release, asymmetric catalysis, and so on). Therefore, the study of the processes of NPs synthesis from acetylene by various methods is of great importance, for instance, in plasma of RF capacitive gas discharge. NPs formed in plasma reactors previously were considered inapplicable in technologies and industries. However, recent findings revealed plasma-assisted synthesis as a prospective approach for the controlled formation of C- and Si-based NPs with high yield.^12−15^ These entities are proposed as a new class of multifunctional nanocarriers for applications ranging from bioactive cargo delivery^12,13^ to reinforcement of 3D-printed polymer structures.^14^

Usually, for the deposition of a polymer film, technological gas-discharge chambers with horizontal electrodes, on which samples or plates are placed,^16−18^ are used. In this geometry, NPs, which are negatively charged and are efficiently retained inside the plasma by the electrostatic force, are formed in the plasma of the gas discharge. If the particles become large enough, then the force of gravity can overcome the electrostatic force and the particles can fall onto the electrode surface and become embedded in the bulk of the growing polymer film. The conditions for confining NPs in the plasma volume with a horizontal arrangement of electrodes are quite well studied,^17−27^ but for the case of vertical electrodes, the data are scarce.^17,28−30^ It is known that, in the high-voltage near-electrode sheath, the electric field is relatively strong, and only sufficiently large particles can leave the plasma. In contrast, with a vertical arrangement of electrodes, a much weaker radial field opposes gravity, and smaller particles can leave the plasma volume and fall onto the dielectric wall of the discharge tube, giving a ground to consider this approach as a reasonable basis for controlled synthesis of NPs with desired median size. In order to elucidate the quantitative regularities of the confinement of NPs in the discharge with vertical electrodes, in this research we experimentally studied the processes of NPs formation in the plasma of an RF capacitive discharge in acetylene, traced the main parameters of the obtained NPs, and also determined theoretically the conditions for the retention of NPs in such a plasma.

## 2 Experimental Section

**Figure 1:**
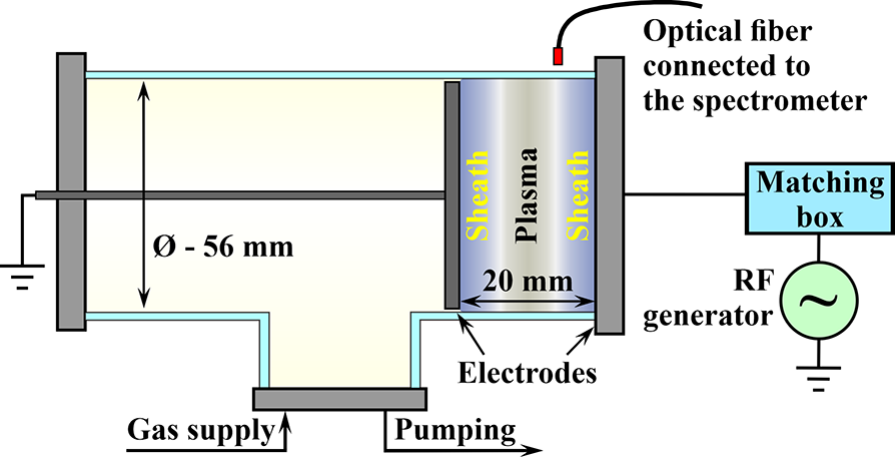
Scheme of the experimental setup. Metal parts are shown in gray; the glass tube is blue colored.

RF capacitive discharge was used to form and study NPs. The schematic diagram of the discharge chamber is shown in Figure 1. The discharge was ignited in a T-shaped glass tube of 56 mm inner diameter. A solid metal flange (potential RF electrode) terminating the horizontal part of the T-tube (on the right in the figure) was supplied with RF voltage from a generator with a frequency of 13.56 MHz. The RF electrode voltage was fixed at 600 V. The second electrode could be moved along the horizontal part of the discharge tube and was grounded. The diameter of this electrode was 55 mm. Both electrodes (potential and grounded) had equal areas contacting with plasma. The electrodes were at a close distance from each other, and there were no closely spaced grounded elements around the chamber. Visually, the discharge was completely symmetrical. To ensure the symmetry of the discharge and remove the self-bias voltage, a choke of 4 mH inductance was additionally connected between the electrodes. Therefore, the parameters of the layers near both electrodes, the forces acting on the nanoparticles in these layers, and the films deposited on the surface of the electrodes are expected to be similar. From the experiment, film samples of coatings from both electrodes had the same properties.

The bottom flange was used to inject and pump out acetylene. During the experiments presented in this paper, the distance between the electrodes was 20 mm. Both electrodes were placed vertically as shown in Figure 1. The studies were carried out at the acetylene pressure of 1 Torr. To measure the gas pressure, a Baratron 627 capacitance manometer (MKS Instruments, USA) of 10 Torr range was used.

The iHR 320 (Horiba, Japan) spectrometer was utilized to study the optical emission spectra of the discharge plasma in acetylene in the wavelength range of 200–1000 nm for gas mixture analysis in the discharge chamber immediately.

During the discharge operation, a polymer film was deposited on the electrodes, on the samples attached to them (glass and stainless steel polished to a mirror finish), and also on the walls of the tube. Besides the film deposited on the surfaces bounding the plasma, NPs were formed in the plasma volume being able to fall down to the walls. Therefore, glass substrates were placed on the discharge tube wall to collect the films and NPs for further comprehensive analysis. The morphological peculiarities of the specimens were preliminary checked via field emission scanning electron microscopy (SEM). A CrossBeam 1540 XB microscope (ZEISS, Germany) operated at 5 down to 2 kV accelerating voltage was employed and an in-lens secondary electrons detector was used for image acquisition. A high-resolution (HR) transmission electron microscopy (TEM) investigation was performed on specimens utilizing a JEM-2200FS (JEOL, Japan) instrument fitted with an in-column Ω-filter, a TemCam-XF416 (TVIPS, Germany) CMOS-based camera and operated at an acceleration voltage of 200 kV. Snapshots were recorded by applying energy filtering. Gatan DigitalMicrograph software was used for HRTEM image processing. To make our samples suitable for TEM study, the film flakes and NPs were placed on Cu microscopic grids with 10 nm thick free-standing a-C films on them.

## 3 Results and Discussion

### 3.1 Experimental Results

#### 3.1.1 Study of Processes in Acetylene Plasma

Usually, researchers apply either a flow-through system (a discharge tube with a constant fresh gas injection on one side and reaction product evacuation on the other) or a closed system (in which, after establishing the required gas pressure, both the inlet and the evacuation lines are closed).^17,30^ Our discharge chamber is intermediate because fresh gas is constantly supplied to the chamber, but there is no direct passage of the entire flow of acetylene through the discharge.

In polymer-forming gases (during the synthesis of the films on the surfaces and NPs in the plasma volume) a sharp decrease in gas pressure is observed immediately after the ignition of the discharge, i.e., the plasma plays the role of a “vacuum pump”.^29,31^ The magnitude of the pressure drop is influenced by the initial pressure of acetylene in the chamber, the gas flow, and the amount of RF power transferred to the plasma. Usually, the pressure in our experiments decreased by a factor of 2–5. The gas entering the discharge gap between the electrodes is almost completely consumed for the formation of polymer films and NPs.

**Figure 2:**
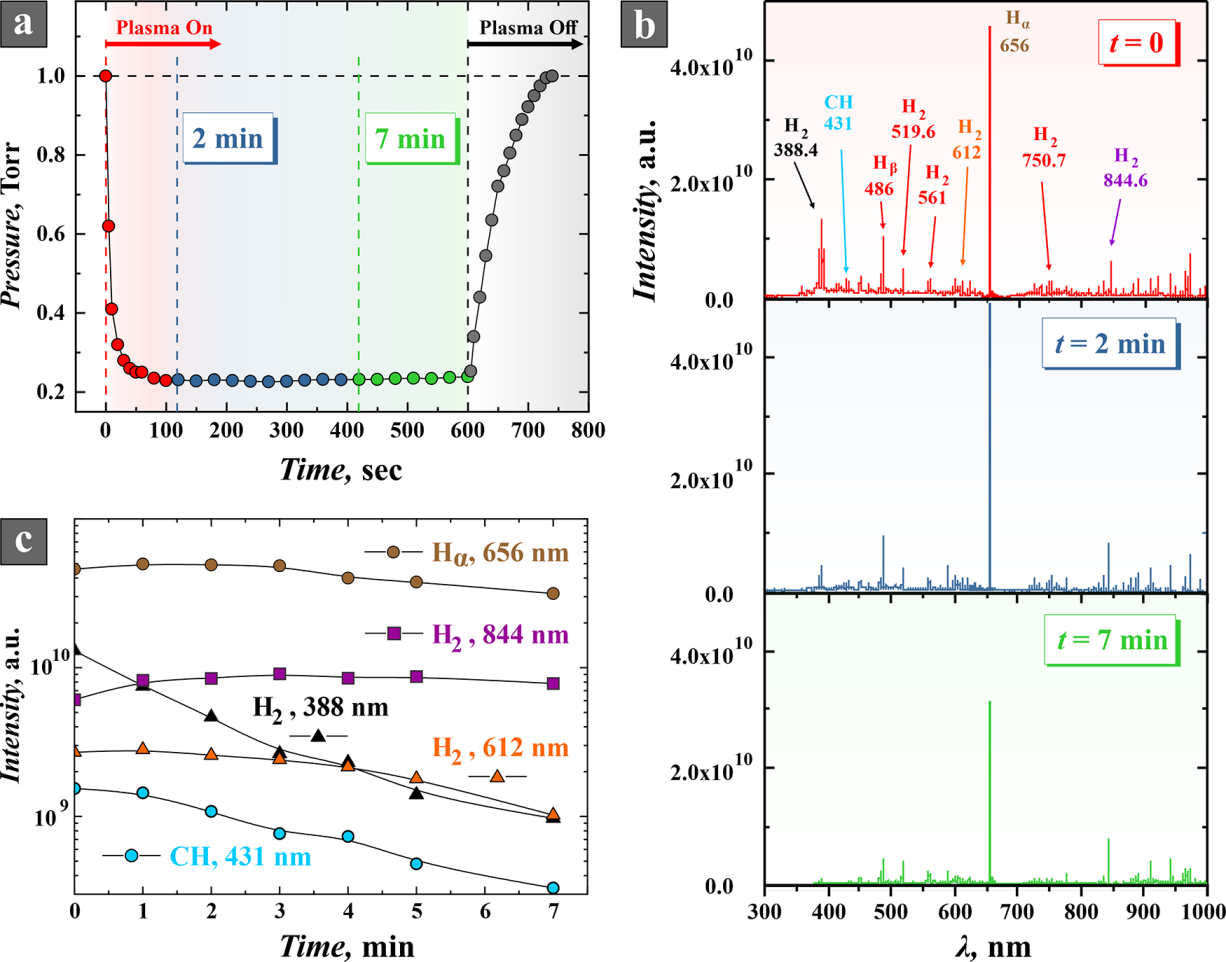
Temporal evolution of gas pressure and optical emission during discharge operation in acetylene. (a) Dependence of the discharge chamber pressure on the operation time. (b) Emission spectra of RF discharge in acetylene immediately after gas breakdown, after 2 and 7 min of discharge burning. (c) Dependences of emission line intensities of atomic and molecular hydrogen, as well as of CH molecules, on the discharge burning time. The initial acetylene pressure was 1 Torr, and RF voltage amplitude was 600 V.

Figure 2 shows the time course of the gas pressure when the discharge is turned on and off. One can see in the figure that the appearance of the plasma reduces the pressure in the chamber by several times compared to the initial one. In this experiment, the inlet gas flow was 2 sccm producing a pressure of 0.05 Torr with a fully open valve of the pumping system. Then the vacuum valve was partially closed until the pressure in the chamber reached a stationary value of 1 Torr. After that, the RF voltage of 600 V was applied to the electrode causing acetylene breakdown. The process of the polymer film deposition on the electrodes and tube walls and the formation of NPs in the plasma volume began simultaneously with the RF discharge ignition. At the same time, acetylene molecules are intensively consumed for the formation of polymer chains with the release of a small amount of hydrogen,^26,27^ which causes a sharp pressure decrease in the chamber (see Figure 2a). Note that hydrogen is evacuated faster from the chamber due to the higher conductance of the output valve for hydrogen, which enhances the “vacuum pump” effect.^29,31^

The following values allow an understanding of the gas pathway in the presented system. After the discharge ignition, the pressure drops by about five times, which means the gas flow to the pump decreases in the same proportion while the gas inflow remains unchanged. Thus, 80% of the input gas flow is absorbed by the polymerization process inside the discharge gap, and we have a constant gas input of 1.6 sccm into the gap through the circular slit around the electrode. The slit conductance can be estimated as 10 L/s with the molecular gas flow (Knudsen number for 0.5 mm slit at 0.2 Torr pressure is 0.6). Thus, the pressure drop between the discharge gap and the main chamber is about 2.5 mTorr or 1.25% of the pressure value. This allows us to consider that the pressure is practically the same in the discharge volume and in the rest of the chamber. Due to this fact, it was possible to connect the pressure sensor through the grounded flange (left in Figure 1). It would be more correct to connect the sensor to the discharge gap immediately, but an RF voltage of 600 V amplitude is applied to the right flange obstructing the correct operation of the sensor.

Let us now consider the dynamics of optical radiation from the RF discharge plasma in acetylene. The RF capacitive discharge consists of two near-electrode sheaths (in which the positive space charge predominates), as well as a quasi-neutral plasma in between them (Figure 1).^32,33^

A quartz optical fiber was connected to the optical spectrometer. The end of the fiber was placed vertically on the top of the tube near the boundary of the near-electrode sheath collecting optical radiation from the upper part of the discharge tube (see Figure 1). This arrangement was chosen because preliminary observations showed that the polymer film is deposited on the upper part of the discharge tube more slowly than on its other parts. In addition, near the boundaries of near-electrode sheaths, the glow was usually brighter in our experiments. In the near-electrode sheaths, it is necessary to distinguish between the anode and cathode parts of the RF period.^34^ During the anode part of the period, when the corresponding electrode has a positive potential and plays a role of an instantaneous anode, electrons fill the near-electrode sheath and partially escape to the anode. However, with time the sign of the potential on the electrode changes to negative, the electrode becomes an instantaneous cathode, and the electrons are swept out of this sheath. In this case, electrons are heated, and they acquire energy sufficient not only for excitation but also for the ionization of gas molecules.^35−38^

The optical emission spectra from the plasma were measured successively at different moments of time after the ignition of the discharge (see Figure 2b). One can see in the optical emission spectra that almost all the glow coming out of the discharge tube is emitted just by hydrogen atoms (Balmer series) and hydrogen molecules with a small addition of CH (which is indicated by the presence of the band with the most intense line at 431 nm). In all our experiments with acetylene, the H_α_ line (656 nm) dominated in the optical spectrum of discharge radiation (see Figure 2b). One could conclude that acetylene almost completely dissociated in the burning RF discharge. However, in the book by Pearse and Gaydon,^39^ it is indicated that excited acetylene molecules emit radiation in the ultraviolet range (with a wavelength of less than 287 nm). Such short-wavelength radiation could not pass through the glass of the discharge tube. Therefore, the absence of acetylene lines in the wavelength range measured by us is not evidence of its complete dissociation.

#### 3.1.2 Microscopic Characterization of the formed NPs and Polymer Films

Analyzing optical spectroscopy data presented in Figure 2b it can be concluded that a significant intensity weakening of the lines belonging to the short-wave (blue) part of the emission spectrum is observed with time during the discharge operation. While the lines of the long-wave part of the spectrum change slightly. The intensity evolution of several lines of hydrogen atoms and molecules with time is shown in Figure 2c. It can be seen that the intensities of the lines in the violet part of the spectrum (for example, the 388 nm line of molecular hydrogen as well as the headline of the CH 431 nm band) monotonically weaken during the discharge. The intensity of the H_2_ 612 nm line (the Fülcher band) in the orange part of the spectrum decreases only slightly over 4 min, but with further discharge burning, the intensity of this line decreases much faster due to the increased thickness of the polymer film deposited on the tube walls. The intensity of the Balmer H_α_ line (656 nm) from the red part of the spectrum during the first minute of the discharge burning increases by about 10% compared to the initial intensity immediately after the ignition of the discharge, then over the next 2 min, its intensity remains stable and only then decreases. The intensity of the 844 nm infrared line of molecular hydrogen increases by about 1.5 times during the first 3 min and then begins to decrease.

Note that polymer films deposited in a pulsed RF discharge in a mixture of argon and acetylene by Zajičkova et al.^40^ have the highest absorption of light in the ultraviolet part of the spectrum, and absorption monotonically decreases with increasing light wavelength without any peaks. However, it must also be taken into account that the intensity of the emission lines depends on the density and temperature of the electrons. Both of these values can change significantly during the burning of the discharge, in which the accumulation and growth of nanoparticles occur. Therefore, Figure 2b and c reflects the simultaneous influence of both processes (the polymer film growth and the change in plasma parameters over time) on the emission of optical radiation from the discharge plasma.

The discharge ignition in acetylene initiates the deposition of a polymer film on the electrodes and chamber walls, as well as the formation of NPs in the plasma bulk. The formation of NPs is actually a process of plasma polymerization, which occurs in the discharge volume rather than on the surfaces of the chamber.^17^ A layer of NPs does not appear on the tube wall immediately after the discharge is ignited since the growth of NPs takes some time.^19−23,41,42^ In the course of our experiments, the lower part of the tube wall began to be covered with a layer of NPs after about 20–40 s of the RF discharge burning. At the same time, the layer of NPs appears only on the lower part of the discharge tube and only within the quasi-neutral plasma (the corresponding photograph can be found in Figure S1 in the Supporting Information). In the near-electrode layers and in the upper parts of the tube wall the tube surface is covered only with a brown polymer film with different thicknesses in different parts of the discharge.

After the discharge was turned off, the chamber was opened, and the obtained NPs and polymer films were studied. The photograph in Figure S1 shows the polymer film deposited on the discharge tube, as well as loose material of the same brown color resting on top of the film on the bottom side of the tube. Upon further analysis, it turned out that the loose material is just the NPs stuck together.

**Figure 3:**
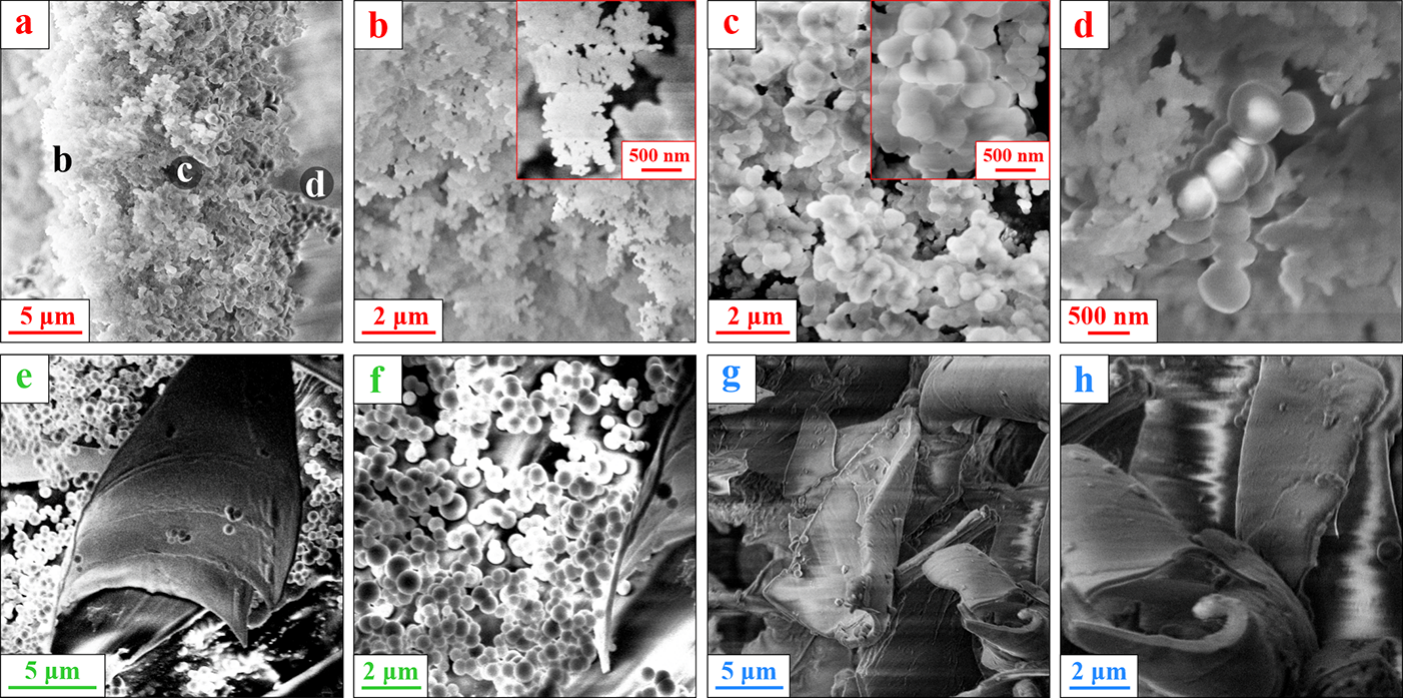
SEM images of (a–d) a flake-like conglomerate of NPs deposited on the tube wall. (a) Cross section of the flake, (b,c) magnified images of the corresponding regions of the flake. (d) “Nanograpes”, a synergy of NPs of >500 nm size from the region (d) of the flake and <100 nm ones from (b) region highlighted in Figure 3a. (e,f) NPs and polymer film chunks collected from the bottom of the tube. (g,h) Polymer film exfoliated from the electrodes (see Figure S2).

**Figure 4:**
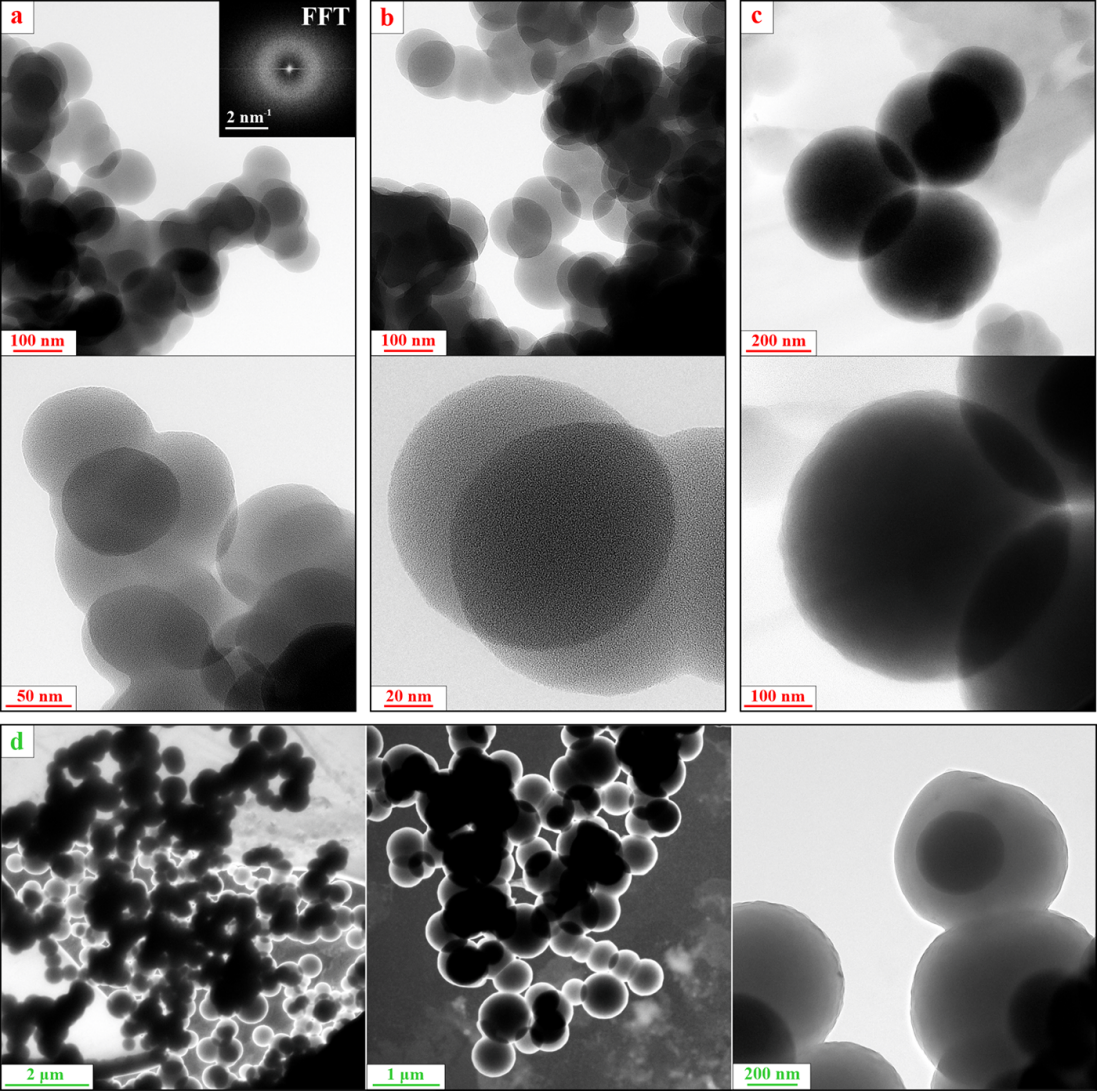
(HR) TEM images of NPs and polymer films collected from the walls of the tube. Zero-loss energy-filtered images of NPs arrays taken from the different regions of the sample: (a) magnified image of the region (b) Figure 3; (b) spherical NPs from the region (c); (c) huge spherical particles from the region (d). Energy-filtered images of the entities typical for the specimen presented in Figure 3e,f. The representative fast Fourier transform (FFT) pattern is shown in Figure 4a (inset).

The results of the morphological SEM investigation of the polymer film together with NPs collected from the different regions of the tube are summarized in Figure 3 while corresponding (HR) TEM images are shown in Figure 4, bringing light to the structural features. In Figure 3a, one can see a representative cross-section image of a flake-like conglomerate of NPs deposited on the tube walls. This flake has a thickness of approximately 14 μm. A closer look at the peculiarities of its through-thickness structure revealed several distinct regions shown in Figure 3b–d. We believe that these features are dealt with various discharge confinement conditions achieved at different stages of synthesis. Unfortunately, the initial orientation of the described flake in relation to the plasma cannot be defined, so the only nonspeculative conclusion that can be made is that we have obtained a mixture of NPs of different sizes with no information on the time of formation of each type of NPs. Region (b) represents a flattened left-hand side of the flake, and its magnified images can be found in Figure 3b (SEM) and further in Figure 4a (TEM). One can see that the specimen structure is the coalesced NPs with a mean diameter of 60–100 nm (radius *a* = 30–50 nm) (Figure 4a). Meanwhile, region (c) in the middle of the flake shows bigger spherical NPs with a diameter of 120–140 nm (radius *a* = 60–70 nm) (see Figures 3c and 4b). Region (d) in the right-hand side rough surface of the flake contains bigger spherical NPs with the diameter varying in a range from 350 to 600 nm (Figures 3d and 4c).

Typical NPs along with chunks of polymer film found on the bottom of the tube closer to the electrodes presented in Figures 3e,f (SEM) and 4d (TEM). One can note spherical NPs with a predominant diameter of 400–600 nm (radius *a* = 200–300 nm) with a minor presence of larger NPs with a diameter of up to 900 nm.

Let us now describe some properties of the polymer film deposited on vertically arranged electrodes. A photograph of the electrode with the deposited film can be found in Figure S2 in the Supporting Information. The film has sufficiently high transparency, as a result, interference rings are formed, which are clearly visible in the photo. Figure 3g,h shows polymer film from the electrodes. The deposited film was delaminated using a sharp blade (thereby, the film was broken into small pieces of arbitrary shape) and subjected to examination. Analysis of the SEM images of the samples from the electrodes shows that all the specimens contain the film pieces only; spherical NPs were not found (Figure 3g,h). Polymer films collected from various regions of the tube (e.g., presented in Figure 3e–h) were thicker than 200 nm, which rules out fruitful TEM imaging. It is noteworthy that according to (HR) TEM data the NPs, as well as regions of polymer films, which were transparent for an electron beam, are amorphous. The representative fast Fourier transform (FFT) pattern is shown in Figure 4a (inset). Additional SE SEM images revealing the surface morphology of the films collected from the tube walls and from the vertical electrodes can be found in the Supporting Information in Figure S3a and b, respectively.

Summarizing the experimental results, numerous NPs can be found in the discharge chamber after RF capacitive discharge burning in acetylene. The vast majority of them are spherical with a diameter of 400–600 nm (radius *a* = 200–300 nm) with a minor presence of larger NPs with a diameter of up to 900 nm and of smaller ones with a diameter of 60–140 nm. It must be emphasized again that the spherical particles, which fall in abundance on the tube wall, have never been found on the electrodes. In the following chapter, using analytical calculations, we will elucidate the regularities of nanoparticle confinement in the plasma of the discharge with vertically positioned electrodes. Utilizing the elaborated theoretical model, we will check the possibility of particles of various sizes reaching the electrodes and compare the outcome with experimental data on the most probable size of observed NPs.

### 3.2 Theoretical Analysis of Nanoparticle Confinement Conditions in Discharge with Vertical Electrodes

It is well-known that small particles in a plasma are negatively charged and can be held in the plasma volume by electrostatic forces. NPs growing in the plasma bulk can leave it upon reaching sufficiently large sizes. A nanoparticle located in the discharge volume can be affected by gravity force, electric field force, ion drag force, thermophoresis force, and neutral drag force.^43−45^ Gravity pulls the nanoparticle down. In the RF capacitive discharge, layers of space charge are present near each electrode, and the plasma has a time-averaged positive potential with respect to the electrodes. Therefore, a negatively charged NP is affected by an electric field force directed from the electrodes toward the plasma. In the opposite direction (versus the electric field force), the ion drag force acts on the NP. Since the average plasma potential has a positive sign,^46^ the positive ions that have come to the sheath boundaries due to diffusion are accelerated by the electric field toward the electrodes. These ions, colliding with the NP, push it from the plasma to the electrodes. If the discharge current flowing through the plasma heats up the neutral gas and a noticeable gas temperature gradient appears, then the thermophoresis force can also act on the NP, pushing it out of the plasma toward colder electrodes. In addition, if gas is blown through the discharge chamber, its directed flow can carry NPs and a neutral drag force arises.

In the case of small NPs with a diameter of tens to hundreds of nm, the gravity, the electric field, and the ion drag forces are dominant.^41,44^ Therefore, in the case of our interest, we can stick to just these forces. The balance of these forces will make it possible to determine which particles can be kept in the plasma and which will leave it. As already mentioned, small particles are effectively held in the bulk of the plasma by electrostatic force, but when they grow to a sufficiently large size, they can leave the plasma.

The main difference between the cases of horizontal and vertical electrodes is that the voltage drop in the near-electrode sheath (its constant component) can reach hundreds and even thousands of volts,^32,33,46^ while the dielectric walls of the discharge tube are under a floating potential relative to the plasma, the sign of which is usually negative, and the value is usually 3–5 electron temperatures and does not exceed 15–20 V.^47,48^ Consequently, only relatively small NPs can be retained in the discharge plasma with vertically arranged electrodes (in the experiments described above, we saw particles with a typical diameter of up to about 400–600 nm), while larger particles must overcome a small electric field force and fall onto the tube wall. With horizontal electrodes, gravity is opposed by a much stronger electric field in the near-electrode sheath, and much larger particles can be retained.

In the following subsections, we analyze in detail the conditions for confining NPs in the strong electric field of the near-electrode sheath and in the weak radial ambipolar field. The near-electrode sheath is explored first, including the derivation of basic equations in one subsection and analysis of NPs retention in the next one. The study of NPs confinement by the ambipolar field follows, starting with the calculation of the ambipolar electric field in the plasma for the specific case of NPs present in plasma. Next, the balance of forces is analyzed, and, finally, the NP retention by the ambipolar field is discussed including a comparison with the experimental results.

#### 3.2.1 Analysis of Processes in the Near-Electrode Sheath of a RF Capacitive Discharge

Let us analyze the forces acting on an NP in the near-electrode sheath of an RF discharge with vertically located electrodes. Since it is important for us to answer the question about the possibility of the NP crossing the sheath, we will limit ourselves to this sheath and will not additionally pay attention to the presheath and the unperturbed quasi-neutral plasma region. Thus, we will focus on the electrostatic force and the ion drag force. Other forces, viscous gas drag, and thermophoresis, usually play a role in the plasma volume, but in the sheath, they are much smaller than the Coulomb force.^49^

**Figure 5:**
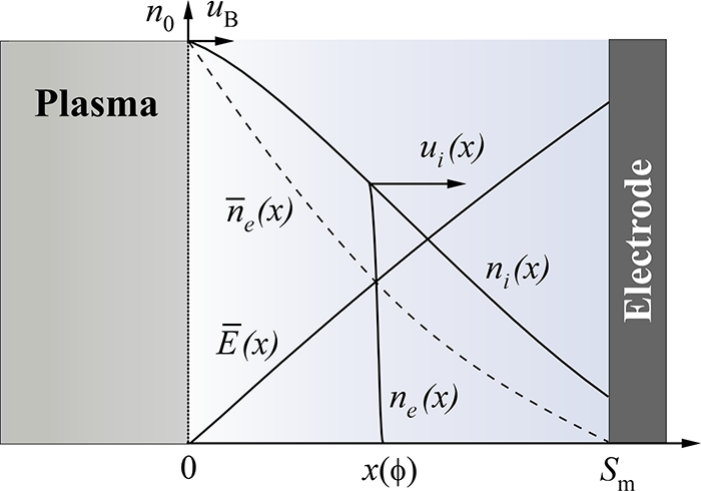
Structure of the near-electrode sheath in the RF discharge. The dependencies shown are the axial distributions of the mean electric field *E̅*(*x*), ion density *n*_i_(*x*), instantaneous *n*_e_(*x*), and average *n̅*_e_(*x*) electron density profiles over the period of the RF oscillation.

Positive ions enter the sheath with the Bohm velocity *V*_B_ = (*kT*_e_/*M*_i_)^0.5^, where *k* is the Boltzmann constant, *T*_e_ is the electron temperature, *M*_i_ is the mass of the ion. The plasma density at the sheath boundary is equal to *n*_0_ (see Figure 5). If the ionization processes in the sheath can be neglected, then the ion current density *J*_i_ remains unchanged, so
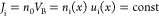
1where *n*_i_(*x*) and *u*_i_(*x*) are the density and velocity of positive ions in the sheath at a distance *x* from its boundary with the presheath. When moving away from the sheath boundary, the ion velocity *u*_i_(*x*) increases due to acceleration in the time-averaged electric field *E̅*(*x*)^50^
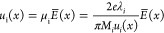
2
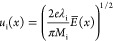
3where μ_i_ and λ_i_ are the mobility and mean free path of ions, respectively. Therefore, the ion density decreases with distance from the sheath boundary according to the law
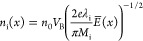
4

The presheath is a quasi-neutral region; the densities of ions and electrons are equal in it. However, a strong violation of quasi-neutrality is observed in the sheath. Since the ions are massive, they do not have time to respond to the instantaneous RF electric field *E* and are able to accelerate only in the time-averaged electric field *E̅*. At the same time, light electrons move in an instantaneous RF electric field *E* and during one-half of the RF period (in the anode phase, when the electrode is an instantaneous anode) gradually fill the sheath, and upon transition to the cathode phase, they are swept by the field from the sheath. Therefore, the negative charge of NPs will be replenished when the sheath is regularly filled with electrons, despite the collisions of NPs with positive ions.

**Figure 6:**
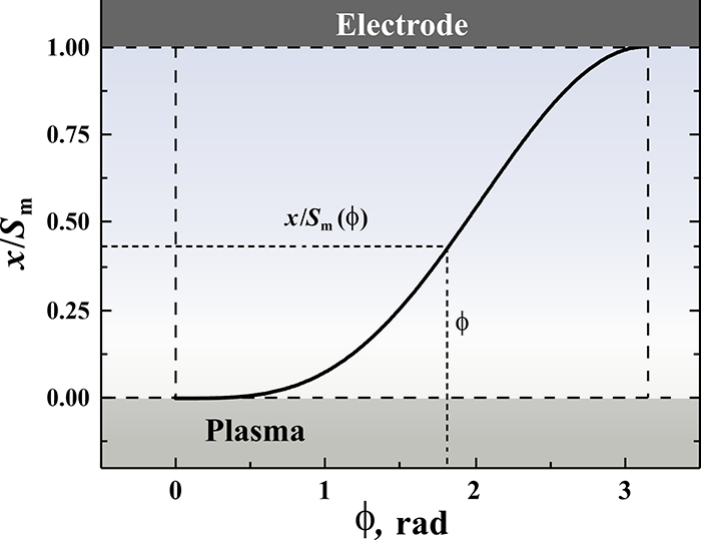
Dependence of the dimensionless coordinate of the boundary of oscillating electrons *x*/*S*_m_ on the phase of the RF field ϕ = ω*t*, calculated using the eq 5.

Since the RF voltage drop across the sheath significantly exceeds the electron temperature, the boundary of the part of the sheath filled with electrons almost does not blur (as schematically shown in Figure 5), and its position *x* is related to the phase of the RF field ϕ = ω*t* by the equation^50^ (see Figure 6)
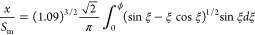
5Here we introduced the maximum sheath thickness *S*_m_. The *x* value changes from 0 (at the minimum electrode voltage) to *S*_m_ (when the electrons were completely swept out of the layer by the strong electric field at the maximum voltage), thus, the dimensionless value *x*/*S*_m_ changes from 0 to 1.

For the convenience of further calculations, this equation can be approximated as follows:
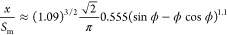
6or
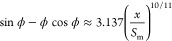
7

Electrons are present in the sheath within a fraction of the RF period, which is related to the phase of the RF field ϕ = ω*t* as 1 – (2ϕ/2π). That is, they are almost always present near the sheath boundary, but they are almost absent near the electrode. Therefore, we will use the formula for the average electron density over the period, obtained in the papers^50,51^
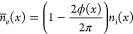
8and RF current density in the sheath is
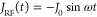
9The current density *J*_0_ is related to the maximum sheath thickness *S*_m_ and the RF voltage drop across it *V*_s_ by the relation^52^
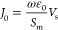
10Since positive ions move in the time-averaged electric field, we need a formula for it, obtained in refs (50) and (51)
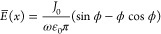
11which we rewrite taking into account eqs 7 and 10 in the form:
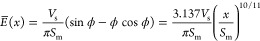
12Now we need to determine the potential and charge acquired by the NP placed in the sheath. For this, we use the formulas for the currents of electrons *I*_e_ and positive ions *I*_i_ given in ref (53). The electron current is related to the floating potential φ_s_ of the NP, the particle radius *a*, and the average electron density *n̅*_e_(*x*):
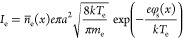
13where  is the mean electron velocity. The positive ion current to the NP is determined using the formula
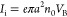
14which, taking into account eq 1, takes the form
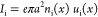
15It should be noted that, in contrast to the case of a dust particle in a quasi-neutral plasma, here we take into account only the directed motion of ions in the sheath, neglecting the focusing of ions in the attractive field of the NP in comparison with the strong electric field of the sheath.

Since the NP is isolated and is under a floating potential φ_s_, the currents of electrons and positive ions to it must be equal to each other. Let us equate eqs 13 and 15, and, taking into account eqs 3, 8, and 12, we obtain the equation that allows calculation of the floating potential *φ*_*s*_ of the NP
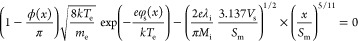
16Knowing the value of the floating potential φ_s_, one can determine the charge *Q* of the NP by the formula for the capacitance *C* = *Q*/φ_s_ = 4πε_0_*a* of an isolated sphere charged to the potential φ_s_^54−61^
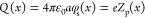
17where *Z*_p_ is the number of electrons attached to the particle. Using the charge *Q*(*x*), one can find the distribution over the near-electrode sheath of the Coulomb force *F*_E_(*x*) acting on the charged NP
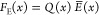
18

Even with a small penetration into the sheath, the ion velocity significantly exceeds the ion sound velocity (*u*_i_ ≫ *V*_B_), which allows us to write the expression for the ion drag force in a simplified form^53^
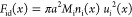
19

To calculate the potential and charge of the NP, we needed to know the mean free path of acetylene ions in their own gas λ_i_. For this, we used the value of the kinetic diameter of the acetylene molecule *d* = 3.3 Å:^62^
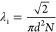
20where *N* is the concentration of gas molecules, while eq 20 takes into account that the mean free path of ions is  times greater than the mean free path of molecules.^51^ Then we have λ_i_[*m*] = 3.51 × 10^–3^/p[Pa] for acetylene ions.

#### 3.2.2 Discussion of NP Retention by the RF Sheath near the Vertically Arranged Electrode

**Figure 7:**
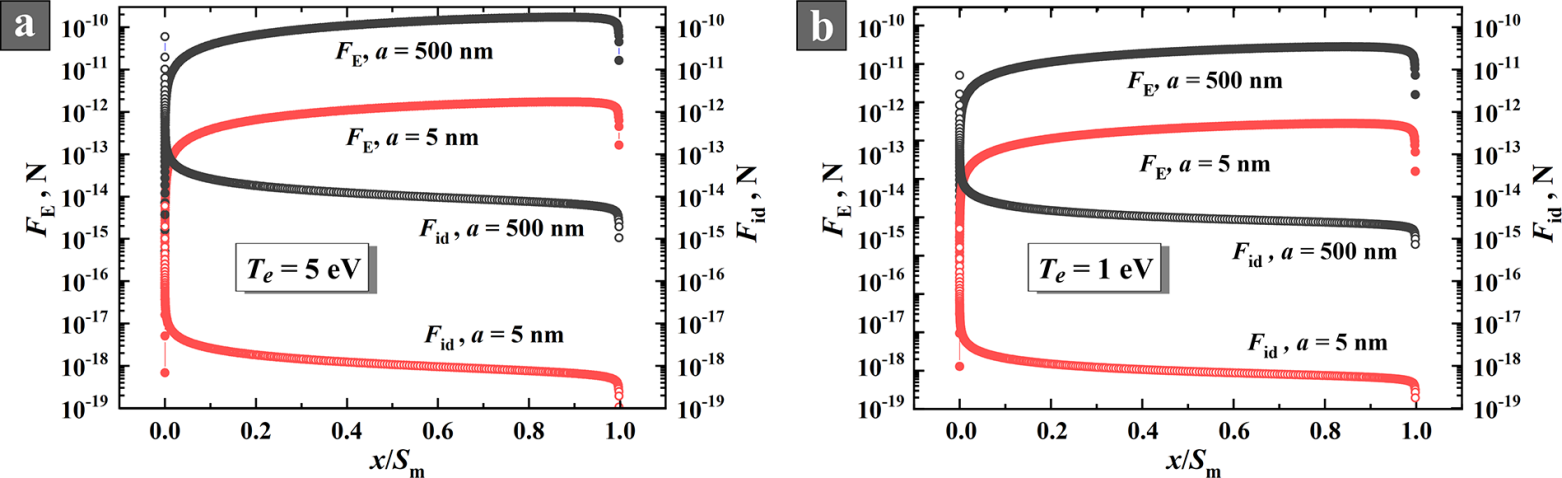
Axial distributions of ion drag force *F*_id_ and Coulomb force *F*_E_ in the sheath for NPs with the radii of 5 and 500 nm for *T*_e_ = 5 eV (a) and *T*_e_ = 1 eV (b).

Using the equations obtained above, we carried out systematic calculations of the forces acting on NPs in the near-electrode sheath of RF capacitive discharge. Now, let us analyze the results of our calculations for the ion drag force *F*_id_ and Coulomb force *F*_E_ across the near-electrode sheath for model particles of two different radii: 5 and 500 nm (see Figure 7). These particle sizes were chosen as limiting values in order to cover all the possible NP sizes according to our experiments. In general, the calculations were carried out in a wide range of nanoparticle sizes (from 1 to 500 nm), gas pressures (0.01–1 Torr), plasma densities (10^8^–10^10^ cm^–3^), and electron temperatures (1–5 eV). Here we present the results for an electron temperature of 5 eV. Note that, in the plasma of RF capacitive discharge (in a low-current α-mode) in an electropositive gas, for example, in argon, the electron temperature *T*_e_ is approximately equal to 1–2 eV.^63−65^ However, the addition of nanoparticles to the plasma may lead to an increase in *T*_e_ up to 3–5 eV.^65^ Furthermore, in the plasmas of electronegative gases (acetylene is one of them^20,26^), the electron temperature can exceed 5 eV.^66^ Therefore, the value of electron temperature *T*_e_ = 5 eV is reasonable. Nevertheless, we performed calculations for different electron temperatures in the range 1–5 eV. We present in the figures below the results for *T*_e_ = 1 eV and *T*_e_ = 5 eV.

From the analysis of the equations, the following conclusions can be drawn. Since factors *a*^2^ were reduced when equating eqs 13 and 15, the potential of an NP does not depend on its radius (within the framework of the assumptions made). In this case, *Z*_p_ is directly proportional to the particle radius *a* (eq 17). Near the sheath boundary (at *x*/*S*_*m*_ → 0), the particle potential *φ*_*s*_ and the number of electrons attached to it *Z*_*p*_ sharply increase, which is apparently related to our assumption that positive ions arrive at the NP as a directed flow and are not focused by the electric field of the charged particle (this approach was used, for example, in ref (67)). But since the purpose of our calculations is to determine whether the NP can cross the entire sheath and reach the electrode, the assumption we have chosen is quite justified. Throughout the sheath, φ_s_ and *Z*_p_ slowly decrease as one approaches the electrode. Note that, near the electrode surface, a sharp decrease in φ_s_ and *Z*_p_ is observed up to the sign reversal. That is, the NP that was negatively charged throughout almost the entire sheath takes on a positive charge near the electrode. According to eq 8, there are few electrons in this region of the sheath, they appear here for a small fraction of the RF period, and, accordingly, more positive ions than electrons now interact with the particle.

The main result of the analysis (Figure 7) is that, near the electrode, where the electric field is maximal, the ion drag force *F*_id_ is 4–6 orders of magnitude less than the Coulomb force *F*_E_ that indicates the fundamental impossibility for NPs to reach the surface of vertically located electrodes. Increasing the particle radius from 5 to 500 nm reduces the ratio between *F*_id_ and *F*_E_ by a factor of 100, which still remains a reliable barrier to the arrival of NPs at the electrode. This result is fully in line with our observations discussed in section 3.2, e.g., Figure 3g,h. Note that only a particle with a radius greater than 20 cm can overcome the ion drag force. Since *F*id ∝ *a*^2^ and *F*_E_ ∝ *a*, these two forces become equal to each other only for the extremely large particles that cannot be grown in a plasma process chamber. It is well-known that very small nanoparticles near the electrodes may be neutral or even positively charged, and these particles would be deposited on the electrode. Our model does not take these particles into account. However, it should be noted that we carefully searched for nanoparticles during TEM and SEM imaging of samples taken from the electrodes, but we never found nanoparticles there. Perhaps the point is that the path of the nanoparticle through the sheath is rather long in time, and the particle, undergoing collisions with electrons and ions, cannot remain positive or neutral long time enough to fly through the entire layer. But if the time-averaged charge of NP is negative, then it will be thrown out of the layer into the plasma.

An abrupt change of the curves at *x*/*S*_m_ → 0 (Figure 7) attracts attention. This result is explained by the fact that, according to eq 12, the electric field at the boundary between the sheath and plasma is formally equal to zero, which is an approximation. The model describes only the sheath and does not take into account the weak field in the plasma. Therefore, the initial segments *F*_id_ and *F*_E_ near the sheath boundary should not be taken into account. The purpose of the described model was to find out whether a charged nanoparticle can cross the entire near-electrode sheath and reach the electrode surface. Since the strongest decelerating field appears near the electrode, a narrow space near the layer boundary does not affect the main result.

#### 3.2.3 Ambipolar Electric Field in the Plasma with NPs

In the case of the near-electrode sheath with a large voltage drop of hundreds to thousands of volts considered above, the electric field force is large, and it is impossible for NPs to overcome it when the electrodes are vertical. However, for NPs falling down onto the wall of a dielectric tube, the Coulomb force opposing the gravity force will be much smaller. The dielectric wall of the discharge tube is under a floating potential with respect to the plasma in contact with it. Charged particles (electrons and ions) arrive in equal amounts per unit area of the tube wall due to ambipolar diffusion.

The ambipolar diffusion in a common plasma consisting of electrons and positive ions is a well-known process.^48^ But in our case, an additional difficulty in calculating the ambipolar field is that NPs present in plasma in large quantities and having acquired a negative charge of tens or even thousands of unit charges can make a significant contribution to the ambipolar diffusion process.

Ambipolar diffusion in a plasma consisting of electrons, positive ions, and charged NPs has been the subject of a number of studies (see, for example, refs (68−70)). In the studies, to find the magnitude of the ambipolar electric field, the balance equations for each type of particle, the Poisson equation, etc. are numerically solved. However, analytical expressions for either the ambipolar field or the ambipolar diffusion coefficients are not given. This makes it difficult to use the results obtained by the authors of refs (68−70). At the same time, for a plasma consisting of electrons, positive, and negative ions, such convenient equations are given in the literature.^71−76^

Here we use the technique for obtaining the ambipolar diffusion coefficients and the ambipolar electric field strength proposed by Thompson in ref (71). We will consider a plasma consisting of electrons, positive ions, and negatively charged NPs. Let us write the expressions for the fluxes of all the particles:
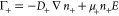
21
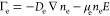
22
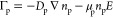
23
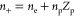
24where *D* and μ are the diffusion coefficient and mobility, *n* is density of charged particles, subscripts “e”, “+”, “p”, refer to electrons, positive ions, and negative NPs, respectively, and *E* is the strength of the electric field. The plasma is assumed to be quasi-neutral: i.e., the total density of positive ions equals the sum of densities of electrons and the number of electrons attached to all NPs per unit plasma volume. We also assume that the total fluxes of positively and negatively charged particles (electrons and NPs) are equal:
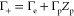
25Let us introduce the dimensionless quantities δ = *n*_p_/*n*_e_ (the ratio of nanoparticle density to electron density), γ = *T*_e_/*T*_+_ (the ratio of electron temperature to the temperature of positive ions), τ = *T*_e_/*T*_p_ (the ratio of electron temperature to nanoparticle temperature). Then, excluding the electric field strength *E* from eqs 21–23 and using eqs 24 and 25, we obtain the following expressions for the flows:
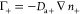
26
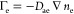
27
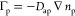
28where the ambipolar diffusion coefficients for positive ions, electrons, and negatively charged NPs are, respectively:
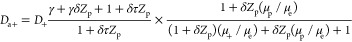
29
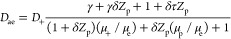
30
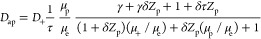
31

The strength of the ambipolar electric field *E* is determined by equating 21 and (26):
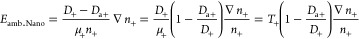
32Here we have used the Einstein relation *T*_+_ = *D*_+_/μ_+_. Substituting (29) into (32), we obtain the expression for the ambipolar electric field:
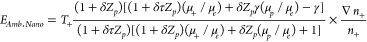
33

Note that, in the absence of NPs, the expression for the ambipolar electric field in plasma (consisting only of electrons and positive ions with equal concentrations *n*_+_ = *n*_*e*_ = *n*) takes the usual form:^48^
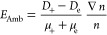
34

#### 3.2.4 Balance of Forces Acting on a Nanoparticle in the Ambipolar Field

Let us now consider the forces acting on a nanoparticle in the radial direction in the plasma volume with the electrodes arranged vertically. In this case, we need to take into account gravity, electric field force and ion drag force. The balance of these forces makes it possible to keep small NPs in the discharge plasma, while sufficiently large NPs can overcome the Coulomb force (under the combined action of gravity and ion drag force) and fall to the bottom of the discharge tube.

We will calculate the Coulomb force both for the usual ambipolar field *E*_Amb_ (eq 34)
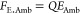
35and for the ambipolar electric field in a plasma with NPs *E*_Amb.Nano_ (eq 33)
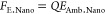
36

Now we need to determine the charge of the nanoparticle *Q*. To do this, we must equate the fluxes of electrons and positive ions to the surface of the nanoparticle, which will allow us to determine its floating potential φ_s_. In the near-electrode sheath (we considered this case above), the directed flow of positive ions hits the nanoparticle. The ions enter the sheath with Bohm velocity and are rapidly accelerated in the strong electric field of the sheath toward the electrode, while electrons periodically fill the sheath and maintain the negative charge of the NP. In the plasma volume, both electrons and positive ions arrive at the NP continuously and from all sides (approximately isotropically). Therefore, we must use other expressions for the currents of electrons and positive ions per NP. For electrons, we can take eq 13 replacing *n*_e_(*x*) in it with *n*_e_:
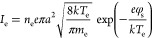
37In this case, the plasma is assumed to be quasi-neutral according to eq 24. In the range of gas pressures studied by us, positive ions arrive at the surface of the NP, experiencing collisions with gas molecules. In ref (77), an expression was proposed for the effective ion current to a NP *I*_i.eff_, which takes into account the cases of weak collisions *I*_i.wc_ and strong collisions *I*_i.sc_:
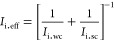
38
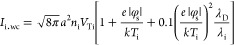
39
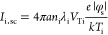
40The charge of the NP was determined by equating eqs 37 and 38 taking into account eqs 39 and 40, and then it was used to calculate the dependence of the Coulomb forces acting on the nanoparticle *F*_E.Amb_ and *F*_E.Nano_.

**Figure 8:**
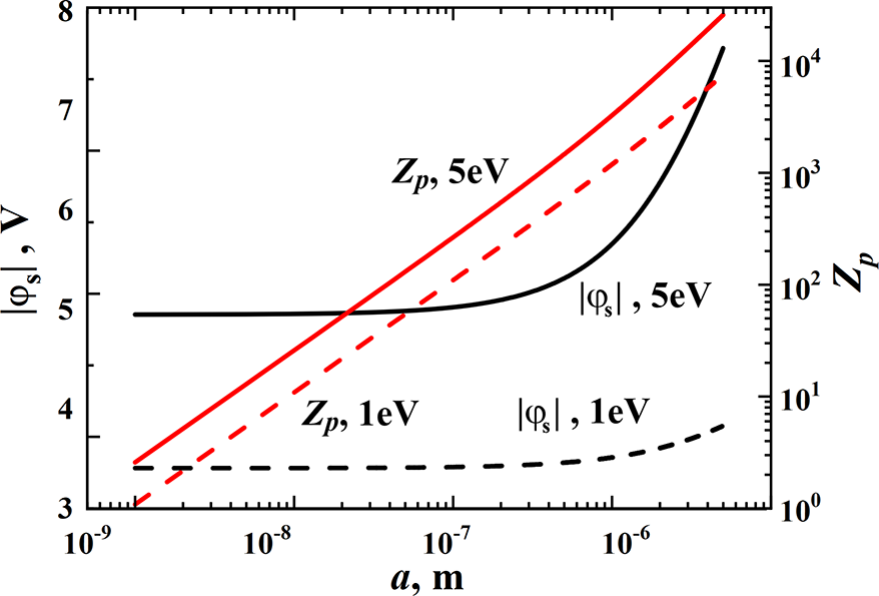
Dependencies of the NP potential φ_s_ and charge *Z*_p_ on the particle radius *a* calculated for the following parameters: *T*_e_ = 5 and 1 eV, *T*_+_ = *T*_p_ = 500 K, γ = τ = 116, *n*_+_ = 10^9^ cm^–3^.

The calculated dependencies of the NP potential and charge on the particle radius are shown in Figure 8. For *a* = 200 nm (particles of such a size are shown in Figure 4), the particle charge is approximately 550 electrons at the electron temperature *T*_e_ = 5 eV (justified above) and the positive ion density *n*_+_ = 10^9^ cm^–3^, which is quite typical for such a kind of plasma.^22^ An electron temperature decrease to 1 eV causes the decrease of the particle charge without qualitative change of the dependencies.

Next, we determine the force of gravity *F*_g_ acting on the nanoparticle
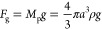
41The density of polymer films deposited in acetylene plasma ranges from 0.4 g/cm^–3^ stated in ref (30) to 0.6 g/cm^–3^ stated in ref (78); therefore, in the calculations, we took the average value 0.5 g/cm^–3^.

When calculating the Coulomb forces acting on the NPs *F*_E.Amb_ and *F*_E.Nano_, we also need to know the nanoparticle mobility μ_p_. We defined it as follows. Since the mobility of a charged nanoparticle
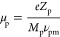
42where *ν*_pm_ is the collision frequency of a nanoparticle with gas molecules, then using the expressions for the nanoparticle mass
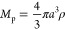
43collision frequency
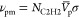
44where σ = π*a*^2^ is the nanoparticle cross section,
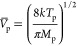
45after simple algebraic transformations we obtain
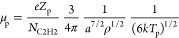
46

Above, we considered the ion drag force acting on a nanoparticle located in the near-electrode sheath, i.e., a region with a strong electric field where the ion velocity can exceed the Bohm velocity. However, in the plasma volume, ions move from the plasma to the wall of the discharge tube under the action of a relatively weak ambipolar electric field. In this case, we used the formula given in ref (70) to calculate the ion drag force
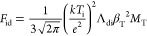
47where β_T_ = |*Q*|*e*^*2*^/(λ_D_*kT*_i_) is thermal scattering parameter, **M**_T_ = *u*_i_/*V*_ti_ is the thermal Mach number, and Λ_di_ is the modified Coulomb logarithm for collisions between nanoparticles and positive ions, .
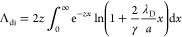
48

#### 3.2.5 Discussion of Nanoparticle Retention by Ambipolar Electric Field

**Figure 9:**
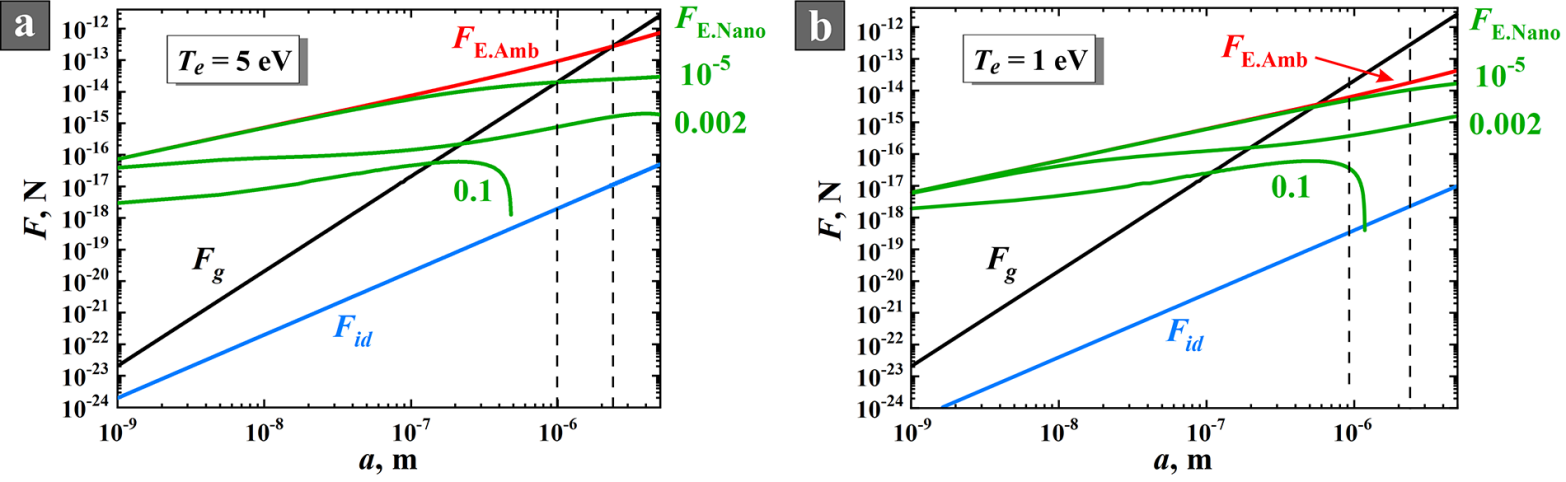
Dependencies of ion drag force *F*_id_, gravity force *F*_g_, Coulomb force in plasma without NPs *F*_E.Amb_ and with NPs *F*_E.Nano_ on nanoparticle radius *a* for different δ (10^–5^, 0.002, and 0.1). Calculated for the following parameters: *T*_e_ = 5 eV (a), *T*_e_ = 1 eV (b), *T*_+_ = *T*_p_ = 500 K, γ = τ = 116, *n*_+_ = 10^9^ cm^–3^.

Using the equations obtained above, we calculated the forces acting on a nanoparticle in radial direction for the plasma bulk region of the RF capacitive discharge with vertical electrodes. The dependencies of ion drag force *F*_id_, gravity force *F*_g_, and Coulomb force (without NPs *F*_E.Amb_ and with NPs *F*_E.Nano_) on nanoparticle radius are shown in Figure 9 for different densities of NPs represented by the parameter δ. It follows from the figure that ion drag force *F*_id_ does not have a significant effect on the nanoparticle movement in a wide range of their radius *a*.

One can see from the figure that the gravity force and the Coulomb force are the growing functions versus the particle size. However, the gravity force grows faster and for any value of δ there is a critical size, after which the particle cannot be confined by the ambipolar field. In a plasma consisting only of positive ions and electrons with *T*_e_ = 5 eV, the Coulomb force *F*_E.Amb_ acting on a nanoparticle balances the gravity force *F*_g_ at *a* ≈ 2.3 μm. However, if there is even a little amount of negatively charged NPs in the plasma, then the Coulomb force decreases and can hold much smaller NPs only. Already at δ = 10^–5^ the critical size decreases more than twice. Figure 9a shows that when δ = 0.002, the equilibrium *F*_E.Nano_ = *F*_*g*_ appears at *a* ≈ 210 nm. NPs of approximately the same size were observed in our experiments (see Figures 3 and 4). If the concentration of NPs is significant, then the Coulomb force further decreases and can even change its direction (see the curve for δ = 0.1 in Figure 9a). The electron temperature decrease to 1 eV (Figure 9b) leads to an insignificant change in the equilibrium particle radius.

The physical reason for the described Coulomb force reduction is the ambipolar field weakening due to the appearance of NPs. In usual plasma, the tube wall is charged negatively versus plasma due to the velocity of negatively charged particles (in this case, electrons) being higher than the velocity of the positive ions. However, in the plasma with NPs, some carriers of negative charge (namely, NPs) are much heavier and much slower than the positive ions. Thus, the plasma-wall potential difference will decrease with the absorption of electrons by NPs (the negative charge carriers become heavier), and even the sign reversal may occur if the NPs density is high enough.

**Figure 10:**
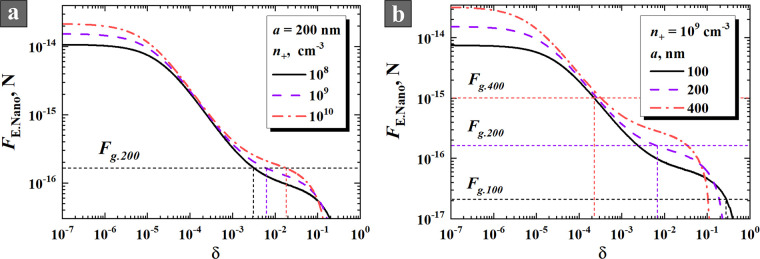
Gravity force *F*_g_ and Coulomb force *F*_E.Nano_ as a function of the ratio of nanoparticle density to electron density δ = *n*_p_/*n*_e_ for different radii of NPs (a) and for different plasma densities (b), calculated for the following parameters: *T*_e_ = 5 eV, *T*_+_ = *T*_p_ = 500 K, γ = τ = 116, *n*_+_ = 10^9^ cm^–3^.

Thus, we can make an important conclusion: the maximum size of the retained nanoparticle decreases with the increase of nanoparticle density. Figure 10 shows this dependence in more detail. Obviously, low concentrations of NPs have little effect on *F*_E.Nano_, but already at δ = 10^–6^, *F*_E.Nano_ begins to noticeably decrease with increasing δ. The equality of *F*_E.Nano_ and *F*_g_ is achieved at different δ for different particle sizes, which is indicated by vertical dotted lines in Figure 10a. NPs with lower radius are reliably held by the ambipolar electric field, while larger particles can overcome the *F*_E.Nano_ and fall to the bottom of the discharge tube. The analogous dependencies for different plasma densities are shown in Figure 10b. The equilibrium value of parameter δ is growing with the plasma density increase.

It should be mentioned that δ, being a key parameter defining the dropping particle size, is a free parameter in our calculations. Unfortunately, our model is not able to predict the equilibrium value of δ. To solve this problem, a complete model of RF discharge with NPs should be developed that extends far beyond the scope of the present research. The importance of the NPs for the discharge operation can be illustrated by the data in Table 1 where the equilibrium value of parameter δ is presented for three different NP size. The last column indicates the parameter  showing the distribution of the negative charge present in plasma between electrons and NPs (by analogy with electronegative plasma where the parameter α represents the degree of electronegativity of the plasma).

**Table 1:** Dependencies of NP Charge *Z*_p_, Relative Density δ = *n*_p_/*n*_e_, and the Parameter α = *Z*_p_δ on the NP Radius^a^

*a* (nm)	*Z*_p_	δ	α = *Z*_p_δ
5	12.9	10.9	140.6
50	130.8	0.92	120.3
100	268	0.28	75
200	558	0.0064	3.57
400	1160	0.00021	0.24

a Calculated for the parameters presented in Figure 10.

One can see that for 400 nm particles the majority of the negative charge is carried just by electrons; thus, the discharge is not disturbed significantly by the presence of NPs in the plasma volume. In contrast, in order to achieve the critical radius of 100 nm, plasma must be highly electronegative. Almost all the electrons in this case are absorbed by the NPs, and the discharge operation is expected to be obstructed. We concluded above that the increase in the number of particles over time should lead to a decrease in the maximum size of retained particles. However, in this case, the particles will intensively absorb electrons from the plasma, which limits the possibility of stable operation of the discharge with too many particles of small diameter.

Thus, one can expect that during the long-term operation of the RF capacitive discharge in acetylene some equilibrium state will be reached with the maximum NP radius of a few hundreds of nanometers. All the smaller NPs grow continuously and, after reaching the critical size, leave the plasma falling down to the tube surface. Thus, the majority of the fallen particles are expected to be of the mentioned critical size that is in good agreement with our experimental results.

One can see in Table 1 that in the nanoscale (that is at *a* < 100 nm) parameter δ is growing in reverse proportion to the particle size; that means the equilibrium for the small particles may be reached only at high NP density. However, due to the decrease of individual charge of each particle, which compensates for the density growth, the parameter α remains around 100 in the whole range of nanometer size. This represents a highly electronegative plasma, but the possibility to reach such values of electronegativity looks feasible. Thus, the applicability of the proposed approach is expected to be extendable toward the nano dimensions.

According to the conclusion made above, the maximum threshold NP size is dependent on the NP density in the plasma. The process of particle formation in plasma is described in detail in ref (41) and begins with the formation and growth of nanoobjects, the size of which can be approximately 2 nm. These nanoobjects are selectively retained in the plasma volume, and when their concentration reaches a critical value of about 10^10^–10^11^ cm^–3^, the phenomenon of fast coagulation of the nanoentities into larger particles occurs. Next, the particles are growing (without significant change of density) up to the critical size, after which they can fall down. In the presented study, we observed continuous deposition of NPs onto the tube wall from the very beginning of the discharge operation, which apparently means that successive generations of nanoparticles can grow and coexist in the plasma.^79,80^ The experiments show that the collected NPs include not only particles of the predominant diameter of 400–600 nm but also smaller ones. According to our current understanding, the observed size dispersion is the result of several nanoparticle generations appearing successively during the 10 min run.

## 4 Conclusion and Outlook

In this work, we investigated the NP formation in the plasma volume of RF capacitive discharge in acetylene with vertically arranged electrodes. It was shown that spherical NPs with a predominant diameter of approximately 400–600 nm can be found on the horizontal tube wall after the discharge operation. NPs of different sizes in the ranges of 60–140 and 600–900 nm were also found in minor quantities. These NPs were held in the plasma volume by the ambipolar field and have fallen on the tube wall when the combined action of gravity and ion drag force was able to overcome the near-wall potential barrier. In contrast, the polymer film is deposited on the vertical electrodes, and no NP was found in the film.

When the RF discharge burns in acetylene, the effect of a “vacuum pump” is observed. Acetylene molecules are effectively incorporated into the deposited polymer film on the electrodes and into the growing NPs in the plasma bulk, while a small amount of hydrogen is released. Therefore, the gas pressure after the ignition of the RF discharge rapidly decreases compared to the initial one. The emission spectra measured near the boundary of the near-electrode sheath on the fly during RF discharge burning contain almost only lines of atomic and molecular hydrogen as well as a CH molecular band with weak intensity. It was also observed that short-wave radiation is strongly absorbed by the grown polymer film, while the lines in the red and infrared parts of the spectrum are weakly absorbed.

To elucidate the possibility of NPs to leave the plasma in the direction of vertically located electrodes, a model of NP retention in a near-electrode sheath of an RF capacitive discharge was built. The floating potential and charge of the particle, as well as the forces acting on it throughout the entire layer, were calculated. It is shown that for the particles of nanometer and micrometer size, the Coulomb force in the layer exceeds the ion drag force by 4–6 orders of magnitude. Therefore, NPs are unable to cross the near-electrode sheath and are pushed out of it back into the plasma.

We also developed a model of ambipolar diffusion of a plasma containing electrons, positive ions, and charged NPs. Analytical expressions are obtained for the ambipolar diffusion coefficients for all three charged plasma components as well as for the strength of the ambipolar electric field. It is shown that NPs with a radius of several micrometers can be retained in plasma by the ambipolar electric field if their concentration is low. However, if the concentration of NPs in the plasma is high and a substantial part of electrons stick to them, then the ambipolar electric field is significantly reduced and can only hold NPs with a radius of a few hundred nanometers. The calculation results agree satisfactorily with our experimental data.

We believe that the described approach to the formation of nanoparticles in the RF discharge with vertically arranged electrodes can be advised as a promising basis for the technology of controlled synthesis of NPs with desired median size. This geometry (compared with the conventional horizontal one) has a prominent advantage allowing the formation of smaller NPs with characteristic sizes from tens to hundreds of nanometers. The developed theoretical model opens the way to conscious control of the size of synthesized particles by fine-tuning the discharge parameters. Nevertheless, our understanding of the effect of discharge parameters on the chemistry of synthesized materials is also still scarce but imperative for both the fruitful implementation of the suggested NP synthesis technique and further improvement of the elaborated theoretical model.

## Data Availability

Raw data that support the findings of this study are available from the corresponding authors upon reasonable request.

## Supporting Information Available

Digital camera photographs of RF discharge in acetylene; the NPs downside and polymer film deposited on the tube surface; the image of the electrode with the deposited film; SEM images showing the surface morphology of the films collected from the tube wall and from the vertical electrode (PDF)
