# Supplementary material for: Synthesis of Nanoparticles and Theoretical Model of Their Retention in Plasma of RF Capacitive Discharge with Vertically Arranged Electrodes in Acetylene
Source: ACS Omega. 2022 Dec 15;7(51):47941–55. doi: 10.1021/acsomega.2c05846 (PMC9798781; doi:10.1021/acsomega.2c05846)
Supplement: Supplementary file 1 — ao2c05846_si_001.pdf [file ao2c05846_si_001.pdf]

SUPPORTING INFORMATION

Synthesis of Nanoparticles and Theoretical Model of their Retention in Plasma of RF Capacitive Discharge with Vertically Arranged Electrodes in Acetylene

Valeriy Lisovskiy[1*], Alexey Minenkov[2†], Stanislav Dudin[1], Sergiy Bogatyrenko[1], Pavel Platonov[1], Vladimir Yegorenkov[1]

[1]*School of Physics and Technology, V.N. Karazin Kharkiv National University, Kharkiv 61022, Ukraine*
[2]*Christian Doppler Laboratory for Nanoscale Phase Transformations, Center for Surface and Nanoanalytics, Johannes Kepler University Linz, Linz 4040, Austria*
*lisovskiy@karazin.ua
†oleksii.minienkov@jku.at

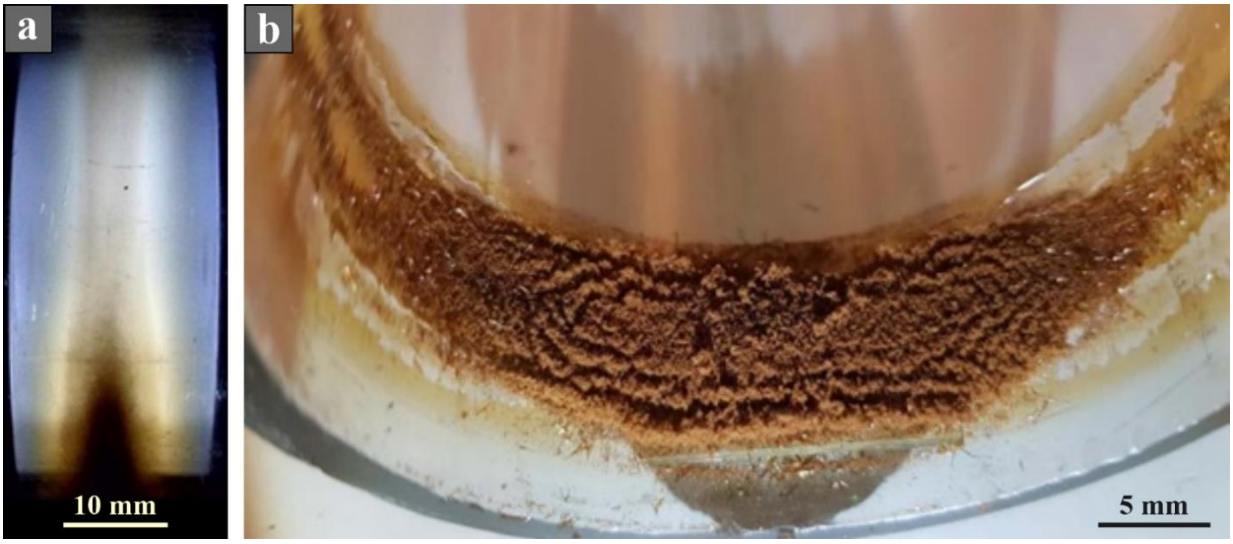

Figure S1. Photos of (a) - the RF discharge in acetylene with NPs deposited on the lower surface of the tube, (b) - the discharge tube after the chamber opening with the layer of NPs downside and polymer film deposited on the tube surface in a single 10-minute run.

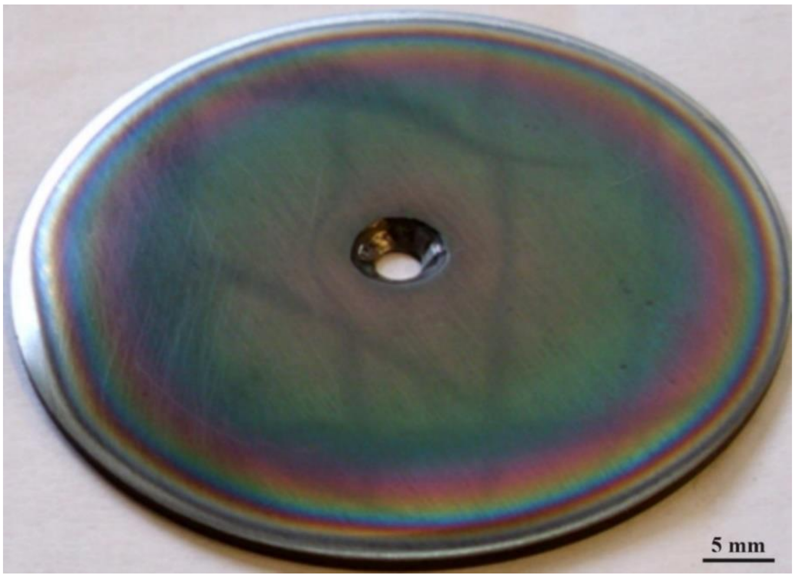

Figure S2. Photograph of the electrode with the deposited film.

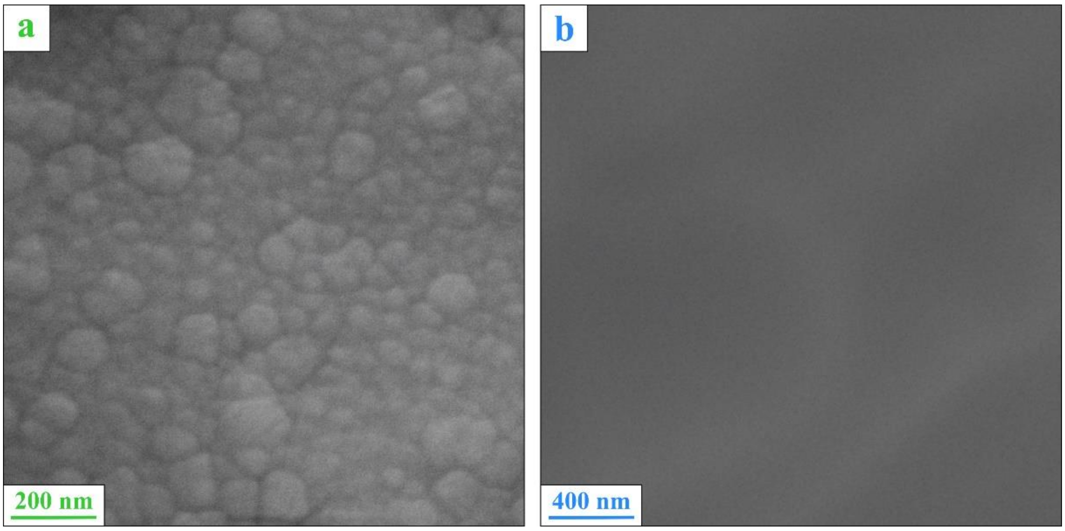

Figure S3. SEM images showing the surface morphology of the films collected from the tube walls (a) and from the vertical electrode (b). The film collected from the wall of the tube together with NPs has a quite rough surface, which points out the incorporation of the falling NPs during the growth. While the film exfoliated from the vertical electrode has a smooth surface without any distinguishable NPs presence.
